# Highlighting the trajectory from intrauterine growth restriction to future obesity

**DOI:** 10.3389/fendo.2022.1041718

**Published:** 2022-11-11

**Authors:** Katherina Vicky Gantenbein, Christina Kanaka-Gantenbein

**Affiliations:** ^1^ Swiss Children’s Rehab, University Children’s Hospital Zurich, Zurich, Switzerland; ^2^ Division of Endocrinology, Diabetes and Metabolism, First Department of Pediatrics Medical School, National and Kapodistrian University of Athens, Aghia Sophia Children’s Hospital, Athens, Greece

**Keywords:** intrauterine growth restriction, IUGR, metabolic syndrome, obesity, offspring, small for gestational age, cardiovascular risk

## Abstract

During the last decades several lines of evidence reported the association of an adverse intrauterine environment, leading to intrauterine restriction, with future disease, such as obesity and metabolic syndrome, both leading to increased cardiovascular and cancer risk. The underlying explanation for this association has firstly been expressed by the Barker’s hypothesis, the “*thrifty phenotype hypothesis*”. According to *this hypothesis*, a fetus facing an adverse intrauterine environment adapts to this environment through a reprogramming of its endocrine-metabolic status, during the crucial window of developmental plasticity to save energy for survival, providing less energy and nutrients to the organs that are not essential for survival. This theory evolved to the concept of the developmental origin of health and disease (DOHaD). Thus, in the setting of an adverse, f. ex. protein restricted intrauterine environment, while the energy is mainly directed to the brain, the peripheral organs, f.ex. the muscles and the liver undergo an adaptation that is expressed through insulin resistance. The adaptation at the hepatic level predisposes to future dyslipidemia, the modifications at the vascular level to endothelial damage and future hypertension and, overall, through the insulin resistance to the development of metabolic syndrome. All these adaptations are suggested to take place through epigenetic modifications of the expression of genes without change of their amino-acid sequence. The epigenetic modifications leading to future obesity and cardiovascular risk are thought to induce appetite dysregulation, promoting food intake and adipogenesis, facilitating obesity development. The epigenetic modifications may even persist into the next generation even though the subsequent generation has not been exposed to an adverse intrauterine environment, a notion defined as the “transgenerational transfer of environmental information”. As a consequence, if the increased public health burden and costs of non-communicable chronic diseases such as obesity, hypertension, metabolic syndrome and type 2 diabetes have to be minimized, special attention should be laid to the healthy lifestyle habits of women of reproductive age, including healthy diet and physical activity to be established long before any pregnancy takes place in order to provide the best conditions for both somatic and mental health of future generations.

## Introduction

### The definition of intrauterine growth restriction

Intrauterine growth restriction (IUGR) is a term attributed to the state of a fetus that was unable to achieve its growth potential due to an adverse intrauterine environment, based on serial fetal ultrasound measurements (1–4). The notion of IUGR should not be confused with the term “small for gestational age” (SGA), that is a descriptive term to characterize a neonate that is born with a birthweight and/or birth length below the -2 SDS for gestational age and sex. Although many neonates are fulfilling the criteria for both IUGR and SGA, it is important to differentiate between these two entities, since the first delineates an adverse intrauterine environment that forced the fetus to a re-adaptation of its metabolic and endocrine determinants, in order to spare energy for survival, that may affect also future growth and development, while the latter term of SGA is not obligatorily the result of an adverse intrauterine environment (2).

According to the expert Consensus of 2016, fetal growth restriction is defined by impaired biometric parameters as well as vascular abnormalities of the placenta such as an increased pulsatile index or an absent end-diastolic flow of the uterine artery (3). The expert Consensus of 2018 (4) proposed following variables as the definition of growth restriction in the newborn: “birth weight under the 3rd percentile or three out of five following points 1. Birth weight under the 10^th^ percentile, 2. Length under the 10^nth^ percentile, 3. Head circumference under the 10^th^ percentile, 4. Prenatal diagnosis of fetal growth restriction, 5. Maternal pregnancy information such as pre-eclampsia or hypertension.”

The current narrative review article aims to highlight the difference between IUGR and SGA, as well as the causes and consequences of IUGR, with special emphasis on the mechanisms linking IUGR to future poor cardiometabolic outcome.

### Differences between intrauterine growth restriction and small for gestational age

It is therefore important to differentiate between the terms fetal growth restriction (FGR) or Intrauterine growth restriction (IUGR) and small for gestational age (SGA). The term SGA defines a fetus whose size is below the 10^th^ percentile for gestational age. Reasons for this can be ethnicity, parental height, maternal weight, or age (5). However, the SGA-fetus may grow along the designated percentiles and there is no pathological condition. In contrast, the term FGR/IURG refers to a pathological condition in which the fetus grows below its expected percentiles. In this case the fetus can also grow over the 10^th^ percentile but does not reach its expected growth potential (6). In other words, a fetus exposed to inadequate protein intake or to increased psychosocial stress of the pregnant woman during gestation may adapt its endocrine-metabolic pathways for survival during the window of developmental plasticity, while a neonate born SGA can merely be born to an otherwise healthy woman of short stature, without being exposed to an adverse intrauterine environment.

### The determinants of fetal growth

#### The adequate placental blood supply

A prerequisite for the appropriate growth of a fetus is the supply of nutrients and oxygen. This is guaranteed *via* the placenta. During pregnancy, the uterine vessels dilate to ensure sufficient blood flow to the placenta. This is regulated by hormones such as estrogens, progesterone, and human chorionic gonadotropin (7). In addition, the placenta grows by the formation of new villi. The number of intraplacental vessels increases so that the fetus is supplied with enough nutrients and oxygen (8). This procedure is regulated by growth factors, such as leukemia-inhibitory factor, epidermal growth factor and vascular endothelial growth factor, which are secreted by the uterine glandular cells (9).

The adequate blood supply through the placenta may therefore guarantee adequate nutrient supply to the fetus. In fetuses with FGR/IUGR the placenta is smaller and the number of villi as well as vessels within these villi are reduced (8). The decreased size of the placenta and the dysfunction of the trophoblasts lead to an undersupply of the fetus with oxygen and nutrients. Moreover, it has been described that both the velocity of the umbilical blood flow as well as the umbilical oxygen delivery are reduced in FGR pregnancies compared to normal pregnancies (10).

Therefore, causes of placental insufficiency may be of vascular origin, such as stenosis or inadequate vascular development/angiogenesis, but also the result of pre-existing chronic disease of the pregnant woman, such as arterial hypertension, predisposing to pre-eclampsia, poorly controlled pre-existing diabetes mellitus, or the influence of toxic agents, such as smoking due to the vasoconstrictive effect of nicotine or substance abuse, in the form of either excess alcohol consumption or drug abuse (7, 8, 11–14).

#### Nutrient supply

Adequate oxygen and nutrient supply through the placenta are therefore of paramount importance for normal fetal growth. During pregnancy, the need for energy increases by 69kcal/day in the first trimester, 266kcal/day in the second trimester and 496kcal/day in the last trimester. The main energy source of the fetus is glucose. Since the fetus cannot produce glucose during intrauterine life, it is dependent on the maternal supply (15). Studies in animals have shown that inappropriate supply of glucose during pregnancy, leading to fetal hypoglycemia, predisposes to growth retardation and to congenital malformations. Intellectual disability due to hypoglycemia has also been well documented as is the case in poorly controlled pregnant women with pre-existing type 1 diabetes mellitus or even gestational diabetes (16). Moreover, an adequate supply of amino acids and omega-3-fatty acids is particularly important for the growth of the fetus and the placenta. In addition, a sufficient intake of iron, iodide, and calcium is essential for normal fetal growth (17). Vitamin D is also indispensable since vitamin D deficiency has been shown to be associated with an increased risk of preeclampsia and the development of gestational diabetes, circumstances that, in turn, affect fetal growth (18). Furthermore, folate supplementation can prevent congenital malformations, especially neural tube defects, preeclampsia or IUGR, caused by folate deficiency. Vitamin Β1 and B6 deficiency are also associated with pre-eclampsia and/or IUGR (19).

#### The hormonal determinants of normal fetal growth

Fetal growth is regulated by hormones mainly secreted by the placenta and the fetus (20, 21). The most important key regulator of fetal growth is fetal insulin, while insulin secreted by the beta-cells of the maternal pancreas does not cross the placenta (22). Thus, the passage of the maternal glucose to the fetal circulation drives the production of insulin by the fetal pancreas, leading to fetal hyperinsulinemia, which, in turn, promotes fetal growth.

During pregnancy, a state of inherent insulin resistance of the pregnant woman, the beta-cells of the maternal pancreas expand to secrete more insulin. In addition to that, the insulin-sensitive organs, such as skeletal muscle and adipose tissue, that in normal circumstances absorb glucose, through the development of this insulin-resistant state, allow more glucose to be available through the placenta to the fetus (23). The glucose of the maternal circulation, therefore, is passively diffused through the placenta to provide adequate fuel for fetal growth. Thus, in situations characterized by hyperglycemia of the mother, such as in case of pre-existing type 1 diabetes or gestational diabetes, the increased glucose induces hypertrophy of the fetal pancreatic beta cells and increased insulin production, leading thus to fetal macrosomia (24, 25). In other words, the paramount role of insulin during fetal development is highlighted by the macrosomia of newborns born to mothers with pre-existing diabetes, where maternal hyperglycemia drives overproduction of insulin by the fetal pancreatic beta cells. On the other hand, in cases of inadequate insulin production by the fetus or inadequate action, such as in the case for example of pancreatic agenesis of the fetus or leprechaunism, a genetic cause of extreme insulin resistance, the newborn is characterized by an extremely reduced birth weight, proving again the paramount role of fetal insulin on fetal growth (26, 27). Furthermore, fetal growth as well as placental development is stimulated by the insulin like growth-factors (IGF), mainly IGF-2 and, to a lesser degree, IGF-1 (28). The role of IGF-2 in fetal growth has been demonstrated in the context of the Silver-Russel syndrome, characterized by extreme intrauterine growth restriction due to disorders in the methylation pattern of the IGF-2 gene in affected fetuses. A further key regulator of fetal growth is the growth hormone variant (GHV) secreted by the placenta. From the 17th week of pregnancy, GHV replaces GH secreted by the pituitary gland in the maternal blood circulation. GHV promotes fetal growth by increasing blood and glucose supply in favor of the fetus (29). Furthermore, fetal growth is regulated by thyroid hormones (30). Thyroid hormones are secreted by both the maternal thyroid gland, especially in the first half of pregnancy, and the fetal thyroid gland, especially in the second half of pregnancy. Thyroid hormones are essential for neurogenesis and osteogenesis of the fetus. During pregnancy, the demand for thyroid hormones and, therefore, also for iodide increases by 20-50%. Consequently, the need-based supplementation of iodide is crucial for pregnant women (31).

All these growth-promoting hormonal factors are counteracted by the glucocorticoids. Glucocorticoids have mainly an inhibitory effect on growth, but are essential for the differentiation of fetal tissues and preparation for extrauterine life (28).

### The causes of impaired fetal growth

Impaired fetal growth may thus be the result either of maternal, fetal, or placental causes (**Table 1**).

**Table 1:** Causes of intrauterine growth restriction.

Causes of Intrauterine Growth Restriction		
*Maternal causes*	Medical conditions	A. Arterial hypertension and Pre-eclampsia B. Poorly controlled Diabetes mellitus C. Hyperthyroidism D. Infections during pregnancy such as rubella, Toxoplasma etc.
	Lifestyle factors	A. Malnutrition B. Smoking C. Substance abuse
*Placental causes*	Inadequate placental blood supply	
	Placental abnormalities	A. Circumvallate placenta B. Placenta previa
	Chromosomal aberrations in the placental tissue	
*Fetal causes*	Genetic causes	A. Chromosomal aberrations B. Monogenic causes C. Imprinting disorders
	Congenital malformations	
	Metabolic causes	
	Twin/multiple pregnancies	
*Varia*		

#### Maternal causes

The most common cause is preeclampsia in the pregnant woman (13). Preeclampsia is defined by maternal hypertension occurring after the 20th week of gestation with proteinuria and/or maternal organ dysfunction and/or placental insufficiency (5). Risk factors for preeclampsia are an antiphospholipid syndrome, history of previous preeclampsia, poorly controlled pre-existing type 1 diabetes mellitus, hypertension, positive family history for preeclampsia, multiple pregnancy, nulliparity, obesity and age over 40y (32). The pathogenesis of preeclampsia is not clearly understood. The hypothesis, however, is that there is an impaired development of the uterine spiral arteries. This leads to an undersupply of the placenta, and consequently, to placental ischemia. This results to the secretion of anti-angiogenic factors into the maternal circulation, which, in turn, induce endothelial damage (33). Preeclampsia is correlated with 5-23% lower birth weight in comparison to uneventful normal pregnancies (34).

A further placental abnormality, a circumvallate placenta is also associated with IUGR (35). In a circumvallate placenta the chorionic surface is smaller than the basal surface, leading to a folding of the membrane margin in an annular shape (35). Circumvallate placenta is associated with persistent vaginal bleeding in the first trimester and premature rupture of the membranes (36). In these cases, the fetus is undersupplied with blood and nutrients, which induces growth restriction. Furthermore, pregnancies with placenta previa are also accompanied by an increased risk for IUGR (37).

Concerning maternal factors leading to impaired fetal growth, inadequate control of maternal diabetes mellitus, as mentioned before, is also a risk factor for fetal growth restriction, as also witnessed by murine studies (38). This is caused by the vasculopathy existing in diabetes, as also observed in humans (39, 40). However, too tight glycemic control in pre-existing diabetes can also lead to growth retardation if the mother has prolonged hypoglycemia, and the fetus does not receive adequate glucose supply (41). On the other hand, a poor glycemic control of maternal diabetes can result to fetal macrosomia due to fetal insulin overproduction as a response to maternal hyperglycemia. The resulting glucose oversupply to the fetus during pregnancy may then be complicated by postnatal hypoglycemia, due to the interruption of the increased glucose supply from the maternal circulation after birth, while the insulin production of the offspring is still stimulated (42, 43). Furthermore, besides impairment of fetal growth, either in the sense of macrosomia or fetal growth restriction, preexisting diabetes mellitus of the pregnant woman correlates with an increased risk of congenital malformations, mainly congenital heart defects, neuronal, musculoskeletal and limb malformations (41, 44).

Another maternal cause of growth retardation is maternal hypertension (45, 46). Fetuses of women presenting gestational hypertension have an increased risk of IUGR as well as an increased risk of fetal morbidity and mortality (47). This can be explained by the vasculopathy and associated ischemia of the placenta, also leading to an oxygen undersupply of the fetus. In addition, gestational hypertension is also associated with a higher cardiovascular risk of the offspring (48).

Furthermore, the lack of micronutrients such as vitamins and minerals can lead to impaired fetal growth and/or congenital malformations (49). Therefore, maternal malnutrition but also restrictive diets in the pregnant woman, as is the case in vegan diets without supplementation of essential micronutrients and vitamins, such as vitamin B12 supplementation, constitute further causes of IUGR (45, 50, 51).

Moreover, impaired fetal growth can be the result of thyroid dysfunction, namely maternal hyperthyroidism (52–54). In studies, including that of Luewan et al., it has been shown that hyperthyroidism and thyrotoxicosis of the pregnant woman increases the risk of growth restriction and low birth weight of the fetus (55, 56). On the other hand, since thyroid hormones are vital for an appropriate fetal development, especially fetal brain development, in a state of thyroid hormone deficiency the fetus cannot develop properly. Hypothyroidism can therefore also lead to impaired growth and development of the fetus (57, 58). Furthermore, it has been observed that maternal hypothyroidism increases the risk of non-reversible intellectual disability of the offspring (31, 59–61).

Infections during pregnancy are also further causes of IUGR. There are numerous causative pathogens such as HIV, Zika virus, Rubellla virus, cytomegalovirus, Toxoplasma gondii, etc. (5).

#### Fetal and genetic causes

Growth retardation can also occur in the context of an underlying genetic disorder. The genetic causes can be classified into chromosomal aberrations (incl. aneuploidy and copy number variants etc.) as well as monogenic causes. Imprinting defects are also a known cause of growth retardation. The most common underlying abnormalities are chromosomal aneuploidies, such as trisomy 13, 18 and 21 (62). The probability of chromosomal aberration increases with the severity of fetal growth restriction (FGR) (63). Copy number variants can also be the cause of FGR (64). In a French multicenter study, a pathogenic or likely pathogenic copy number variant was detected in 7.5% of fetuses with isolated growth retardation diagnosed prenatally (65). Frequent copy number variants associated with FGR are the 22q11.2 duplication, the Xp22.3 deletion as well as the 7q11.23 deletion (66, 67). The 22q11.2 duplication, also called DiGeorge syndrome, is a disorder characterized by immunodeficiency, hypoparathyroidism, and congenital heart disease. Other features can be developmental delay, hypothyroidism, renal as well as skeletal abnormalities (68). Patients with a Xp22.3 deletion suffer from ichthyosis and can also have intellectual disability (69). The 7q11.23 deletion leads to the clinical phenotype of the Wiliams-Beuren syndrome. Williams-Beuren syndrome is characterized by a vascular stenosis, cardiac valve abnormalities, hypercalcemia, renal abnormalities, hypodontia and developmental delay (70). Monogenic disorders can also constitute causes of FGR. Examples of monogenic diseases associated with FGR are Cornelia de Lange syndrome, Smith Lemli Opitz syndrome, Bloom syndrome, 3M, Seckel syndrome (62). Furthermore, imprinting defects can be associated with FGR. Epigenetic modifications can lead to a silencing of certain alleles, so that only one allele, the paternal or maternal one, is expressed in a tissue-specific manner. This process is called genomic imprinting. Defects of this imprinting procedure can lead to imprinting disorders resulting in IUGR, as for example Silver-Russel syndrome (71). In most patients with Silver-Russel-syndrome there is a hypomethylation of the imprinting cluster region on the paternal chromosome 11p15.5, which leads to biallelic silencing of the IGF2-gene and a biallelic expression of the noncoding region H19. It can also be caused by maternal uniparental disomy of chromosome 7 (UPD 7) or other rarer molecular genetic causes. Silver-Russel syndrome is mainly characterized by FGR, postnatal growth restriction, body asymmetry and often developmental delay (72). Further imprinting disorders associated with IUGR are the Temple syndrome, the Kagami-Ogata syndrome, the Prader-Willi syndrome, the pseudohypoparathyroidism 1b and others (73).

In addition, a correlation between FGR and chromosomal aberrations in placental tissue has been described. A chromosomal aberration affecting only one cell line of the placenta, in which the fetus has a regular number of chromosomes, is called “confined placental mosaicism” (CPM). As presented in the review of Eggenhuizen et al., 71.7% of CPM cases resulted in FGR (74).

## The consequences of being born IUGR

### Short term consequences

There are both short- and long-term consequences of being born with IUGR concerning both somatic and mental health as highlighted below and listed in **Table 2**.

**Table 2:** Consequences of intrauterine growth restriction.

Consequences of Intrauterine Growth Restriction		
*Short term consequences*	Increased risk of morbidity and mortality	
	Hypoglycemia	
	Hypothermia	
	Hyperviscosity	
	Respiratory complications	
*Long term consequences*	Metabolic derangements	Metabolic syndrome
		Cardiovascular disease
	Growth impairment	
	Endocrine disorders	Precocious adrenarche
		Polycystic ovarian syndrome
	Nephrological problems	Renal insufficiency
		Hypertension
	Cancer risk	Hepatoblastoma
		Retinoblastoma

IUGR can be associated with complications during the neonatal period. Fetuses born with IUGR have an increased risk of morbidity and mortality. They are also at increased risk of developing hypoglycemia shortly after birth due to reduced glycogen and fat stores and limited ability of gluconeogenesis as well as fat oxidation (75). Moreover, they are prone to hypothermia due to lack of subcutaneous brown fat, disproportionate body mass and increased transdermal temperature loss (76).

Moreover, in the context of placental insufficiency, the fetuses often grow under chronic hypoxia. This leads to increased erythropoiesis resulting in high hematocrit values and subsequent hyperviscosity of the blood. This can result in acute neonatal adverse events such as necrotizing enterocolitis or thrombosis (5). Furthermore, neonates born IUGR are at increased risk for developing respiratory complications (77).

Besides the short-term complications of newborns born IUGR during the neonatal period, much more attention has been laid to the long-term consequences of inadequate fetal growth, in later life, as presented below.

### Long term consequences

#### Adverse future outcomes concerning somatic health and disease

##### Metabolic derangements – cardiovascular risk

During the last decades numerous epidemiological studies have reported an association of being born IUGR with future non-communicable diseases in adolescence or adult life, namely an increased incidence of insulin resistance expressed as future obesity and metabolic syndrome with high risk of lipid abnormalities, endothelial dysfunction leading to arterial hypertension, fatty liver disease, glucose intolerance or even type 2 diabetes in adult life, all contributing to higher cardiovascular risk (75, 78, 79).

Furthermore, it has been reported that the increased risk of metabolic syndrome and cardiovascular disease exists not only during adulthood but also during childhood (78, 79). This phenomenon is accentuated in case of a rapid weight gain during infancy (80). The redistribution of weight gain in favor of abdominal fat mass accumulation takes place mainly between the 2nd and 4th year of life. At the age of 4 years, IUGR children show higher fat mass, insulin resistance and proinflammatory parameters (81). Furthermore, they present an increased risk of dyslipidemia and metabolic syndrome (5, 82). For example, Tenhola et al. studied a group of 55 children with low birth weight and 55 children with appropriate birth weight and found that children born with low birth weight were at increased risk of hypercholesterolemia. Predisposing factors were the female gender, poor catch up growth in height and early initiation of puberty (83).

##### Somatic growth- catch-up growth

Infants born with IUGR usually show a fast growth in the first years of life, called catch-up growth. This is accelerated in the first months of life and continues with a modest acceleration till the 7^th^ year, although most IUGR born children are expected to present catch-up growth and enter the normal trajectories of weight and height until the age of 3-4 years (5, 6, 41). A study by de Ridder et al. showed that 91% of children born SGA reached a normal height during the catch-up growth phase (84). Children that cannot reach the normal height trajectory after their 4^th^ anniversary are less probable to enter the normal height trajectories later. Moreover, studies in infants of diabetic mothers with nephropathy born with IUGR showed that in childhood they had lower height and weight even after the catch up-period (85).

##### Reproductive axis

Low birth weight is associated with insulin resistance and decreased IGF1 levels reminiscent of a state of multi-hormonal resistance (86). This hormonal constellation correlates with increased LH levels and reduced SHBG levels in prepubertal girls pointing to an underlying hormonal setting of PCOS-like phenotype. This may result to precocious adrenarche and increased androgen availability. The increased androgen levels in girls born IUGR predispose thus to the development of polycystic ovary syndrome (87), while no association has been described between low birth weight and disorders of adrenarche or puberty in male subjects (88). This association of former SGA with reproductive axis disturbance and functional hyperandrogenism in girls has been extensively studied by Lourdes Ibanez and Francis de Zegher in previous years (81, 89, 90). However, concerning pubertal initiation, most children born SGA, enter puberty slightly earlier but still in the normal range compared to children born with normal weight for gestational age, so called Adequate for Gestational Age (91).

##### Nephrological problems

A further complication of IUGR is kidney disease. According to the review of Ritz et al. IUGR can lead to a reduced number of nephrons. A low number of nephrons predisposes to glomerular hypertrophy as well as tubular dysfunction, increasing the risk of developing hypertension in later life (92). This is also supported by the Brenner’s hypothesis, according to which a low nephron number is associated with hypertension (93). Furthermore, IUGR is associated with an increased risk of impaired renal function and later development of end-stage renal disease (94–96).

##### Cancer risk

IUGR also appears to be a risk factor for the development of pediatric tumors. According to Spector et al. low birth weight strongly correlates with the risk of developing hepatoblastoma (97). Furthermore, low birth weight is associated with gliomas, with an odds ratio of 2.13 (95% CI: 0.71-6.39 for birth weight <1500g) as well as retinoblastomas with an odds ratio of 2.43 (95% CI: 1.00-5.89 for birth weight <1500g) (98). However, O’Neill et al. postulated that there is no correlation between birth weight and the risk of developing retinoblastoma (99). In addition, it is worth mentioning that high birth weight is also associated with higher tumor risk, namely with an increased risk of leukemia (99).

#### The etiological pathway highlighting the link between IUGR and impaired somatic future outcomes

The initial observations that intrauterine growth restriction can adversely influence health in adult life came from Hertfordshire in the UK, where former IUGR-born babies have been reported to have a significantly increased risk to develop Metabolic syndrome in later life in comparison to those born adequate for gestational age or even large for gestational age (LGA). These initial observations have been reported by David Barker in the late 80ies and early 90ies, who supported the notion of ‘fetal origin of adult disease” and explained the reported association through the “Thrifty phenotype hypothesis” (100, 101).

##### The thrifty phenotype hypothesis

According to the thrifty phenotype hypothesis of Hales and Barker, the fetus in a nutrient-restricted environment redistributes energy for survival mainly directing nutrients supply to the vital organs such as the brain, which leads to an undersupply of other organs such as the pancreas. This brain-sparing effect prioritizes the energy supply to the brain and ensures fetal survival. However, it inevitably leads to a disturbance of insulin homeostasis by provoking insulin resistance of the peripheral organs such as the liver and the muscles. This insulin resistant state predisposes the individual to the development of metabolic syndrome and all its parameters such as dyslipidemia, fatty liver, arterial hypertension and type 2 diabetes mellitus later in life (102).

These initial observations from Hertfordshire, UK, have also been confirmed from data originated from the Dutch famine, where it became clear that children, whose mothers have been undernourished during pregnancy due to the famine developed arterial hypertension in later life, data also confirmed in other parts of the world (103).

##### The notion of developmental mismatches

In other words, in case of impaired intrauterine milieu, for example in the context of protein-restricted maternal nutrition during pregnancy, exaggerated stress of the pregnant woman, inadequate placental nutrient supply or blood underperfusion, the endocrine-metabolic modifications that took place during the important window of developmental plasticity ascertain offspring’s survival on the short term, a notion that is known as Immediate adaptive response (IAR) (104). This also persist into the extrauterine life and confer an increased cardiometabolic risk to the offspring, especially when the restricted intrauterine milieu does not match to the nutrient-abundant extrauterine milieu, as Gluckman supported in his notion of the match-mismatch principle (105, 106) or otherwise reported as predictive adaptive response (PAR) (107).

##### The predictive-adaptive response

According to the notion of predictive adaptive response (PAR) as formulated by Gluckman et al, if the fetus has been exposed to an adverse intrauterine environment, that has induced an immediate adaptive response for survival, during the window of developmental plasticity, this adaptation has prepared the fetus to face the extrauterine environment through a prediction of an also nutrient restricted extrauterine environment. However, if the intrauterine nutrient-restricted environment does not really match to the extrauterine environment, since the nutrient-restricted intrauterine environment is followed by a nutrient- or calories abundant extrauterine environment, then this predictive adaptive response may have long-lasting consequences for future health and disease, supporting the notion of Developmental Origin of health and disease (DOHaD) (108, 109).

##### Developmental origin of health and disease - epigenetic modifications

According to the Developmental Origin of Health and Disease (DOHaD) concept, this increased future cardiometabolic risk of offspring born as IUGR is attributed to epigenetic modifications taking place during the crucial window of developmental plasticity in intrauterine life (109–111).

The probability of the occurrence of metabolic diseases during life can be therefore influenced by prenatal events. Adversaries in the prenatal environment can influence the metabolism of the fetus. These processes can be mainly caused by epigenetic modifications. Epigenetic modifications include DNA methylation and histone modifications (111) that take place already prenatally. During early embryogenesis, the methylation patterns of the fetus are programmed and can be influenced by various factors, such as maternal nutrition (112). As Waterland et al. have shown in a murine model, maternal nutrition affects the phenotype of the fetus by modifying methylation pattern in the offspring (113). These methylation modifications induced by the maternal diet can thus increase the risk of developing metabolic diseases in the offspring (114), which may explain the increased incidence of metabolic syndrome and type 2 diabetes mellitus in IUGR born individuals. Moreover, factors such as maternal nutrition, hypoxia or other pathologies can alter the expression profile of amino acid transporters. This adaptive change in the expression of amino acid transporters in the trophoblast also regulates fetal growth (115, 116).

##### The transgenerational transfer of environmental clues

According to Gluckman et al., these epigenetic modifications that took place in the first generation that has been exposed to an adverse intrauterine environment may even be transferred and expressed in the offspring of the next generation, although this offspring has not been exposed to an adverse intrauterine milieu, suggesting the notion of transgenerational transfer of epigenetic modifications and highlighting the impact of avoiding intrauterine adversaries in one generation to ascertain a healthy outcome of future generations (104).

It is also worth mentioning that this transgenerational effect that has been reported for the female line of inheritance has also an impact from the paternal side affecting not only the immediate subsequent generation, but also the generation after that. Moreover, epigenetic modifications can take place not only during intrauterine life but also during adolescence. Therefore, not only the mother’s nutrition and lifestyle are relevant for the health of the child, but also the grandparents’ nutrition and lifestyle choices (117). In other words, the epigenetic changes apply not only to the maternal epigenome but also to the paternal (118). In this context it is important mentioning that the Avon Longitudinal Study of Parents and Children (ALSPAC), after appropriate adjustment, has demonstrated that early paternal smoking is associated with greater body mass index (BMI) at 9 years in sons, but not in daughters. Sex-specific effects have also been shown in the Overkalix data reporting that paternal grandfather’s food supply was only linked to the mortality relative risk (RR) of grandsons, while paternal grandmother’s food supply was only associated with the granddaughters’ mortality RR. These transgenerational effects were observed with exposure during the slow growth period (concerning both grandparents) of fetal/infant life (grandmothers) but not during either grandparent’s puberty. The authors concluded that sex-specific, male-line transgenerational responses exist in humans and have hypothesized that these transmissions are mediated by the sex chromosomes, X and Y. Such responses add an entirely new dimension to the study of gene-environment interactions in development and health and provide more data concerning the impact of healthy lifestyle choices through the lifespan for future generations (119–121).

##### Molecular mechanisms linking IUGR to insulin resistance and future obesity/disturbed appetite

As mentioned above, children born IUGR are prone to develop insulin resistance in later life (122). This was also demonstrated in a murine IUGR model (123). Long et al. proposed that this observation could be explained by an impaired LRP6-Wnt-signaling pathway, which regulates the expression of insulin -receptors, leading to insulin resistance (124). A further possible underlying mechanism for the insulin resistance in individuals born with IUGR is the upregulation of ACSL1 expression. ACSL1 is a gene which regulates lipid metabolism. The authors claim that the upregulation of ACSL1 could facilitate the catch-up growth. However, this could also lead to increased secretion of esterified fatty acids, which promote insulin resistance and dyslipidemia (125).

Animal studies investigating the link between IUGR and adipogenesis have demonstrated that the underlying mechanisms predisposing to future obesity include a dysregulation of appetite/satiety signals and abnormal adipogenesis (126). According to Ross and Desai (126) there is a developmental origin of adipogenesis and disturbed appetite signals in intra-uterine-restricted newborns. As observed in animal models of IUGR, maternal calorie restriction or ligation of the uterine artery led to increased adult adipogenesis, accentuated when the IUGR status has been followed by a rapid extrauterine catch-up growth. It has been demonstrated that gestational nutrient restriction led to a dysregulation of orexigenic neuronal circuits at the hypothalamic level. The predominant appetite regulatory site, the hypothalamic Arcuate nucleus (ARC) receive signals from peripheral circuits, such as the gastrointestinal tract, pancreas, and the adipocytes but also from central inputs such as the brain. The ARC contains the medial orexigenic neurons (NPY and Agouti-related peptide neurons) and the lateral anorexigenic neurons, the Pro-opiomelanocortin (POMC) and the Cocaine and amphetamine regulated transcripts (CART). During fetal development the hypothalamic neuronal stem cells (NSC) proliferate and ultimately differentiate into neurons. Among those, the ones destined to the ARC appetite center further differentiate to express either orexigenic or anorexigenic peptides. In their experimental animal setting the researchers have demonstrated that intrauterine growth restricted animals resulted in significantly increased food intake with resulting hyperphagia due to dysregulated satiety signals, as evidenced by reduced satiety signals to leptin or, on the contrary, increased responses to appetite stimulatory signals from ghrelin. Moreover, laboratory studies from the same research group have demonstrated that IUGR male offspring have upregulated adipogenic signaling cascade evidenced by an increased expression of enzymes promoting adipocyte lipid storage and synthesis. IUGR adipocytes in culture retained this adipogenetic characteristics even when deprived from the hormonal milieu in which the IUGR offspring has been exposed *in utero* (126). It has therefore been postulated that the mechanisms that result in offspring obesity include the programming of the hypothalamic appetite pathway and adipogenic signals regulating lipogenesis. Processes include nutrient sensors, epigenetic modifications, and alterations in stem cell precursors of both appetite/satiety neurons and adipocytes which are modulated to potentiate offspring obesity.

Furthermore, in a more recent experimental study Gong et al. investigated the Bone marrow mesenchymal stem cells (BMSC) of the intrauterine growth-restricted rat offspring and demonstrated that they exhibited an enhanced adipogenic molecular profile at miRNA, mRNA and protein levels, with an overall up-regulated PPARγ (miR-30d, miR-103, PPARγ, C/EPBα, ADRP, LPL, SREBP1), but down-regulated Wnt (LRP5, LEF-1, β-catenin, ZNF521 and RUNX2) signaling profile (127).

Further experimental data point towards a sex-dimorphic impact of IUGR on future adipogenesis with male offspring exhibiting stronger adipogenic propensity than females, especially with advancing age, also highlighting both the sex dimorphism of such an effect but also the permissive effect of postnatal caloric intake on future obesity development (128).

The disturbance of the hypothalamic-pituitary axis could also predispose to an increased cardiovascular risk. Individuals born SGA show GH resistance, witnessed by increased GH and reduced IGF1 and IGFBP3 levels. Since reduced IGF1 levels are associated with increased cardiovascular risk, this could constitute a further underlying mechanism linking IUGR with increased future obesity and cardiovascular risk (129).

#### Adverse future outcomes concerning neurocognitive health and disease

According to the meta-analysis by Sacchi et al. (2), IUGR is also associated with cognitive impairment. Children born IUGR or SGA have lower cognitive scores than those born AGA. This is the case for both preterm and term babies (2). These cognitive abnormalities can also be verified by functional MRI (fMRI) studies, that have shown reduced para-hippocampal activity in SGA children compared to AGA children (130). Furthermore, children born preterm with IUGR show impaired fine and gross motor function and an increased risk of developing autistic traits in comparison to preterm children born AGA (131). Cognitive impairments are also described in children born with IUGR during their school years, namely presenting learning difficulties with reduced memory and concentration skills. The risk of developing cerebral palsy is also increased in children born IUGR (1). These observations could be explained by the reduced brain volume, observed in children born IUGR (132). In addition, thyroid dysfunction in IUGR children could also contribute to the cognitive impairment (5).

#### The etiological pathway highlighting the link between IUGR and impaired neurocognitive future outcomes

##### The notion of developmental plasticity

Children with low birth weight show neurocognitive abnormalities as described above. This could be explained by disturbed prenatal neuronal development. St. Pierre et al. were able to demonstrate in a murine IUGR model that IUGR is associated with impaired synaptic plasticity in the hippocampus (133). In addition, Brown et al. described that IUGR mice show a reduced number of neural stem cells in the hippocampus as well as a disturbed induction of neuronal differentiation. These processes could be caused by a downregulation of the Wnt pathway (134). Other parameters such as neuroinflammation, a disturbed blood-brain barrier and oxidative stress can also contribute to the pathogenesis of neuronal dysfunction (135).

Therefore, although future neurocognitive outcome is besides the scope of the current review article, these observations of impaired neurocognitive development in case of intrauterine restriction further highlight the importance of an optimal intrauterine milieu to ascertain all aspects of future health.

## Treatment modalities

### The role of breast feeding

Breastfeeding has beneficiary effects on the health of the child. Studies have shown that breastfed children showed a slower weight gain during the catch-up period and a reduced risk of obesity and hypertonia (17, 136, 137). According to the meta-analysis of Qiao et al. breast feeding is associated with decreased risk of childhood obesity. This positive effect also increases with increasing duration of breastfeeding (138). A positive effect of breastfeeding has also been described for children’s cognitive performance (139). Belfort et al. studied 1224 3-years-old and 1037 7-years-old children and found that children with a longer breastfeeding duration showed higher language comprehension scores at age 3 and higher verbal and nonverbal IQ scores at age 7 (140). Furthermore, breastfeeding can have protective effects against necrotizing enterocolitis, an inflammatory disorder common in premature and IUGR neonates (141).

### Appropriate nutrition during infancy, childhood, and adolescence

Optimal maternal nutrition not only during pregnancy but also before pregnancy is of paramount importance for adequate nutrient supply to the fetus, as already presented in the previous sections of this review. During the last years it became moreover clear that the first 1000 days of life starting from conception until the end of the second year of life are critical for both future health and neurodevelopment. In other words, these 1000 days spanning the period of the 270 days of pregnancy plus the 365 days of the first year of life plus the next 365 days of the second year of life play a major role in future health outcome of the offspring (142). A lot of evidence has been accumulated for the importance of the 270 days of pregnancy in terms of healthy nutrition (micro- and macronutrient composition) and avoidance of noxious agents such as tobacco or alcohol abuse in the lifestyle pattern of the pregnant woman. Since special dietetic preferences have also been adopted by young women of reproductive age in modern societies, such as vegan or vegetarian diets that cannot cover micronutrients requirements of the pregnant woman, and several deficiencies emerge as a consequence of such diets like iron, folate of vitamin B12 deficiency, special attention should be laid to supplementation with micronutrients and vitamins, especially vitamin B12 in women following vegan diets (50, 143). Also, the change in lifestyle in modern societies with increasing indoor activities and decreasing sun exposure leads to higher rates of vitamin D deficiency among women. Supplementation of vitamin D3 during pregnancy leads to a decreased risk of preeclampsia and IUGR (144). Furthermore, since deficiency of maternal iron, calcium, magnesium, and selenium is associated with low birth weight/IUGR, the supplementation of these micronutrients in the pregnant woman can be beneficial for the proper development of the fetus (19).

Moreover, moving into the early extrauterine feeding environment, exclusive breastfeeding for the first 6 months of extrauterine life, as already mentioned, should be advocated to diminish the rates of future obesity and other negative health issues. After the first 6 months of extrauterine life, when exclusive breastfeeding can no more completely cover the nutritional needs of the infant, then complementary food should be introduced. It has been demonstrated that initiation of solid foods before the age of 4 months is associated with an increased risk for future obesity. Thus, promoting exclusive breastfeeding during the first 6 months of extrauterine life and avoiding the early introduction of solid foods before the age of 4 months during infancy are important components in the combat against obesity. The time until the completion of the second year of life is important for being exposed to new tasty, low fat, rich in fruits and vegetables nutrition, the so-called Mediterranean diet (145). Thus, promoting healthy food choices not only during infancy but also during childhood are main determinants of the strategy to prevent future obesity, as already reported by the importance of the first 1000 days for future metabolic health, especially when combined with physical activity and avoidance of sleep deprivation (142, 146, 147).

Nutrition also plays an important role during the rest of life, which can be partly explained by epigenetic modifications taking place also at later stages of life, mainly during adolescence. Therefore, epigenetic modifications are important during the lifespan both pre- and postnatally as well as during adolescence. According to Han et al. BMI and smoking during adolescence can influence the DNA methylation (148). Furthermore, studies have shown that exercise and weight loss can also change the methylation of certain genes and thus their expression (149–151). Besides nutrition, further environmental factors such as exposure to chemicals or metals can induce epigenetic modifications (152). Taken together, a balanced diet and a healthy lifestyle during adolescence are of paramount importance for preventing future non-communicable diseases of the current as well as the next generation (17).

### Growth hormone treatment

Children born with SGA who do not show catch up growth up to the chronological age of 4 years are often treated with growth hormone (GH) therapy. The GH administration may start at 2 years of age according to the US Food and Drug Administration (FDA) and at 4 years of age according to the European Medicines Agency (EMA). Beginning the therapy at a young age has beneficial effects on the growth gain (153). For example, Al Shaikh et al. studied retrospectively the growth parameters of 26 patients with SGA. The patients received growth hormone replacement therapy at a dose of 0.025-0.05 mg/kg/day. After 3.5 years of therapy, they observed an increase in height of 1.46 SDS (154). Besides the improvement of growth parameters, GH administration has a favorable effect on metabolism as it reduces the risk of hypertension (155) and leads to a reduction in adipose tissue and lipids at the beginning of the treatment (156). At the end of therapy, individuals after GH treatment have similar amounts of adipose tissue and lower lipid levels compared to untreated ones (87, 157). SGA individuals who have received GH therapy exhibit bone-mineral density deficiencies shortly after cessation of therapy, but these normalize 5 years after cessation of therapy (158). Furthermore, GH treatment has positive effects on the kidney development since it leads to an increased renal length and volume. Moreover, after cessation of GH treatment the renal function has been shown to be comparable between SGA patients who received GH and healthy controls (159).

### Metformin

Another therapeutic target is the dysfunctional metabolism in low-birth-weight children. As described above, IUGR children tend to be overweight and develop metabolic syndrome and type 2 diabetes mellitus. Besides healthy lifestyle habits and healthy nutrition there are not many therapeutic possibilities. Therapeutically, metformin could be considered as a therapeutic option in children born with IUGR, although metformin is neither FDA- nor EMA approved for use in children. A randomized controlled trial by Diaz et al. showed that children treated with metformin had a lower weight and BMI than the control group. There was also an improvement in biochemical variables, with a reduction in glucose and triglyceride levels as well as fat mass (160). Furthermore, studies performed by Ibáñez et al. showed that treatment of low-birth-weight pubertal girls led to reduced fat gain, delayed pubertal development, and improved biochemical parameters (161–163).

Moreover, according to Garcia-Conteras et al., based on animal studies, maternal therapy with metformin could theoretically also be beneficial for fetal growth (164). In the study by Garcia-Conteras et al. IUGR pregnancies modelled by malnourished pigs were investigated. They were able to demonstrate that the weight of the internal organs and the brain was higher in the metformin-treated group than in the control group. Therefore, the authors claimed that metformin therapy of the pregnant woman could contribute to the prevention of IUGR (165). However, the results of such animal studies cannot safely be extrapolated to humans yet.

## Preventive measures

In order to eliminate some of the risk factors for IUGR development, a healthy lifestyle of the woman before and during pregnancy is mandatory. Preeclampsia and gestational hypertension are associated with a higher BMI of the woman. Therefore, healthy diet and regular moderate exercise are recommended (166). According to Crovetto et al., in pregnancies with an increased risk of SGA, following a Mediterranean diet can lead to a risk reduction of SGA births (167). Furthermore, an adequate supply of micronutrients can also reduce the risk of growth restriction. If sufficient coverage is not provided by adequate food intake, such as is the case in restrictive diets, like vegan or vegetarian diets, then supplementation with iron, magnesium, folate, and iodine could be considered (166, 168). In the case of gestational hypertension which cannot be regulated by lifestyle modification, drug therapy is recommended. Methyldopa, calcium channel inhibitors and beta-blockers are allowed for pregnant women, while ACE inhibitors or AT1 antagonists are contraindicated during pregnancy (169). In addition, in pregnant women with increased risk of preeclampsia a low-dose aspirin treatment is indicated. A Doppler velocimetry screening of the uterine artery in the second trimester is also recommended in women with an increased risk of preeclampsia (170). As already reported, smoking, alcohol, and substance abuse are also associated with an intrauterine growth restriction (171–173). Therefore, education of young women of reproductive age to avoid such noxious agents is of paramount importance. However, if the young women used to adopt such unhealthy habits, then their consumption should be ceased long before pregnancy planning and conception.

## Conclusion

There is accumulated evidence during the last decades pointing to the importance of an optimal intrauterine environment to provide the best chances for future health.

On the contrary, according to the developmental origin of health and disease, the susceptibility for future disease greatly depends on the developmental window, during which an adverse environmental cue, leading to intrauterine growth restriction, took place, making an individual more vulnerable to adverse future outcomes such as obesity, metabolic syndrome, hypertension, non-alcoholic fatty liver disease and type 2 diabetes mellitus. All these non-communicable diseases are interconnected ultimately leading to increased cardiovascular risk and mortality.

Moreover, it has been proven that intrauterine growth restriction may generally impair future health, increasing the risk for nephrological problems or even cancer risk. Furthermore, an adverse intrauterine environment leading to IUGR is further associated with future neurocognitive impairments of the offspring.

As a consequence, if the increased public health burden and costs of non-communicable chronic diseases but also mental and neurocognitive impairments have to be minimized, special attention should be laid to the healthy lifestyle habits of young people, especially women of reproductive age, including both avoidance of noxious agents, such as smoking or alcohol consumption, healthy diet and physical activity to be established long before any pregnancy is programmed or takes place and these healthy lifestyle patterns should be sustained through the whole duration of pregnancy in order to provide the best basis for both somatic and mental health of future generations. Furthermore, promotion of breastfeeding and healthy eating habits in infancy, childhood, and adolescence along with physical activity may further minimize the risk of future disease.

## Author contributions

Both authors KVG and CK-G have a significant contribution to the manuscript, have read and approved the final version of the manuscript that has been submitted and affirm that no part of the present manuscript has been submitted elsewhere for publication. In detail, KVG has prepared the initial draft of the ms, has performed the extended literature search on the topic and provided suggestions on the presentation of the specific segments of the ms. CK-G has prepared the outline of the review article and has edited the manuscript. Both authors contributed to the article and approved the submitted version.

## Conflict of interest

The authors declare that the research was conducted in the absence of any commercial or financial relationships that could be construed as a potential conflict of interest.

## Publisher’s note

All claims expressed in this article are solely those of the authors and do not necessarily represent those of their affiliated organizations, or those of the publisher, the editors and the reviewers. Any product that may be evaluated in this article, or claim that may be made by its manufacturer, is not guaranteed or endorsed by the publisher.
